# Antibody-based sensing of pesticide residues and mycotoxins in plant-derived foods: A narrative review of ELISA, lateral-flow immunoassays and immunosensors

**DOI:** 10.1016/j.fochx.2026.104530

**Published:** 2026-10-08

**Authors:** Harsh Kumar, Shivani Guleria, Lukas Peter, Nahid Akhtar, Rabia Asghar, Hasnita Binti Che Harun, Dinesh Kumar, Kamil Kuča, Ayaz Mukarram Shaikh, Endre Harsányi, Béla Kovács

**Affiliations:** a Faculty of Materials Science and Technology, VSB-Technical University of Ostrava, 70800 Ostrava-Poruba, Czech Republic; b Department of Agriculture, Faculty of Agro Based Industry, Universiti Malaysia Kelantan*,* Jeli 17600, Malaysia; c TIFAC-Centre of Relevance and Excellence, Department of Biotechnology*,* Thapar Institute of Engineering and Technology*,* Patiala 147004*,* Punjab*,* India; d Department of Microbiology, Graphic Era (Deemed to be University), Dehradun 248002*,* Uttarakhand*,* India; e School of Medical Technology*,* Beijing Institute of Technology*,* Beijing 100081*,* China; f School of Bioengineering & Food Technology*,* Shoolini University of Biotechnology and Management Sciences*,* Solan 173229*,* Himachal Pradesh*,* India; g Centre for Basic and Applied Research*,* University of Hradec Kralove*,* 50003 Hradec Kralove*,* Czech Republic; h Faculty of Agriculture, Food Science & Environmental Management, Institute of Food Science, University of Debrecen, Böszörményi út 138, Debrecen 4032, Hungary; i Faculty of Agriculture, Food Science, and Environmental Management, Agricultural Research Institutes and Academic Farming (AKIT)*,* University of Debrecen*,* Böszörményi út 138*,* Debrecen 4032*,* Hungary

**Keywords:** Biological macromolecules, Food contaminants, Food safety, Human health, Point-of-need detection

## Abstract

Cereals and their products, as well as fruits and vegetables, form a major constituent of the human diet worldwide. These food items can become contaminated with mycotoxins during farming, transportation, or storage. They are exposed to pesticides mostly during cultivation and also during storage and transportation, where pesticides are applied to prevent pest infestation. Exposure to pesticides and mycotoxins can have detrimental health effects on humans. Hence, there is an urgent need for analytical techniques for rapid, sensitive, and on-site detection of pesticides and mycotoxins in food items. In this regard, antibody-based sensing platforms can be an alternative to traditional adulterant detection technology. The review delineates the issues that affect the quality of fruits, vegetables, cereals, and cereal-based products and can harm consumer health. Furthermore, the review provides a comprehensive overview of antibody-based sensing methods such as enzyme-linked immunosorbent assay (ELISA), lateral flow immunoassay (LFIA), and immunosensors for detecting mycotoxins and pesticide residues in the food matrices. The review suggests that competitive ELISA and LFIA formats were the most extensively investigated approaches for detecting adulterants and contaminants. Among immunosensor-based methods, the electrochemical immunosensors demonstrated the most effectiveness, followed by optical immunosensors.

## Highlights

• Pesticide residues and mycotoxins in the food commodities are critical health hazards.
• Antibody-sensing technologies act as rapid screening tests.
• Nanomaterials integration enhances the sensitivity levels of antibody-sensing platforms.

## Graphical abstract

**Figure:**
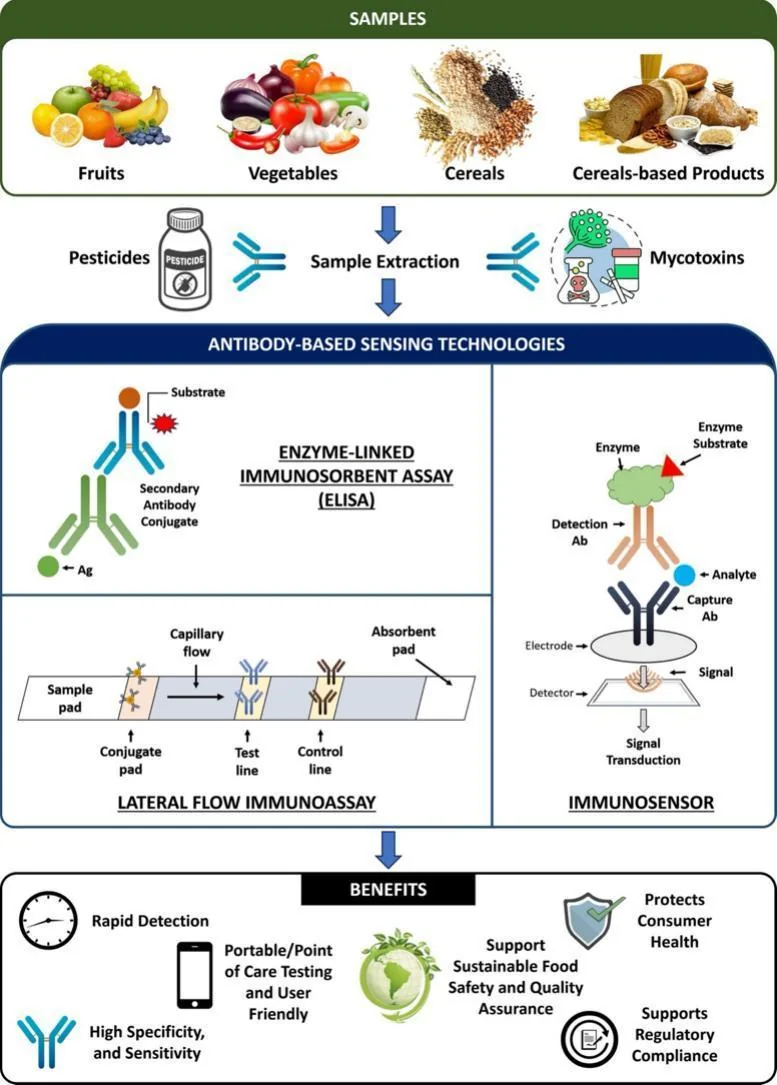

## 1 Introduction

Over the years, consumers have become aware of the important role of proper nutrition and diet in preventing different metabolic and chronic diseases (Mazzoni et al., 2021). Fruits and vegetables are essential components of a healthy diet because they provide a wide range of nutrients, such as vitamins C and A, minerals and electrolytes, fiber, and various phytochemicals with pharmacological properties like antioxidant and antimicrobial activities (Slavin & Lloyd, 2012). Multiple studies have been conducted throughout the world to elucidate the phytochemical profiles of a wide variety of vegetables and fruits. Such studies have helped in identifying various health-promoting bioactive phytochemicals and revealed their modes of action and pathways associated with disease-preventive and health-promoting effects (Mazzoni et al., 2021). Like vegetables and fruits, cereals and various cereal-based products are major constituents of the human diet worldwide. Cereals like rice, wheat and maize consist mainly of carbohydrates like starch, followed by proteins and a relatively low amount of fats (Silva et al., 2020). Hence, cereals are the major source of energy and nutrients and are also important for global food security (Laskowski et al., 2019).

The Codex Alimentarius Commission, jointly established by the Food and Agriculture Organization (FAO) and World Health Organization (WHO) to formulate guidelines and standards for ensuring food safety, defines contaminants as “substances that have not been intentionally added to food or feed and may pose a risk to animal and human health” (FAO, 2026). Similarly, other international agencies such as the European Food Safety Authority and the U.S. Food and Drug Administration (FDA) have described contaminants as unintentionally added physical, chemical, or biological agents in food and beverages that pose a risk to human health and require monitoring and risk assessment (Bereda, 2025). Some of the common food contaminants are toxins, allergens, pesticides, microbes, heavy metals, dyes, and packaging materials that can enter food during processing, production, handling, transportation, and storage. Other sources of the contaminants can be environmental pollution, farming practices, or intentional addition to increase profit margins (Rodriguez et al., 2021). The WHO has stated that consuming adulterated and contaminated food can have detrimental effects on public health and the economy throughout the world. WHO has estimated that nearly 600 million people get sick after consuming food contaminated with chemicals or pathogens, and about four hundred thousand people lose their lives due to the consumption of unsafe foods (WHO, 2024).

According to the Environmental Protection Agency (EPA), pesticides are chemicals applied to control, repel, prevent, or kill plants or animals considered pests. Furthermore, the EPA also considers substances that are employed as plant desiccants, defoliants, regulators, or nitrogen stabilizers as pesticides (US EPA, 2014). Pesticides help improve crop quality and yield, protect crops from plant pathogens and pests, and increase the shelf life of agricultural products (Saad et al., 2025). It has been reported that only about 0.1% of pesticides reach their intended targets, whereas the majority of the pesticides enter the environment and contaminate the air, water, and soil (Chaudhary et al., 2024). Pesticides enter the food chain through contaminated air, water, and soil and can harm human and animal health, as well as ecosystems (Akhtar & Mannan, 2020). Another major group of food contaminants is mycotoxins, secondary metabolites secreted by fungi belonging to *Alternaria* spp., *Aspergillus* spp., *Fusarium* spp., and *Penicillium* spp., and found in major nuts, cereals, and cereal-based products that can have adverse effects on health. Around 400 types of mycotoxins, such as aflatoxins, fumonisins, zearalenone, and ochratoxin, can contaminate crops and nuts during pre- and postharvest stages, as well as during transportation and storage (Niaz et al., 2025). It has been estimated by the FAO that nearly 25% of food crops are contaminated with mycotoxins. However, other reports suggest mycotoxin contamination is much higher, in the range of 60–80%, which may be attributed to advances in the sensitivity of analytical devices (Eskola et al., 2020). Humans can be exposed to mycotoxins through the consumption of contaminated plant-derived foods or indirectly through animal-based products obtained from livestock fed with mycotoxin-contaminated feeds (Al-Jaal et al., 2019; Arce-López et al., 2020). Mycotoxin exposure may also occur through inhalation and skin contact, especially among waste-handling workers and farmers who may encounter mycotoxin-contaminated dust during processing, milling, or harvesting, as well as through inhalation of contaminated indoor air (Al Hallak et al., 2023; Marcelloni et al., 2024). Mycotoxins are capable of causing acute and chronic toxicity in animals as well as humans (Sweeney & Dobson, 1998). By using the search strings “food and pesticide residues” and “food and mycotoxins”, total 8691 and 8469 research papers respectively were retrieved from the Web of Science Core Collection (WOSCC) and choropleths maps were created as shown in Fig. 1A and Fig. 1B. As shown in Fig. 1A, the maximum research published by China followed by USA and Spain, whereas in Fig. 1B, shown the maximum research output was given by China followed by USA and Italy. Fig. 1C and Fig. 1D show the annual number of published documents and the cumulative volume of research articles related to “food and pesticide residues” and “food and mycotoxins”, indexed in WOSCC from 1991 to 2025, respectively.

**Fig. 1:**
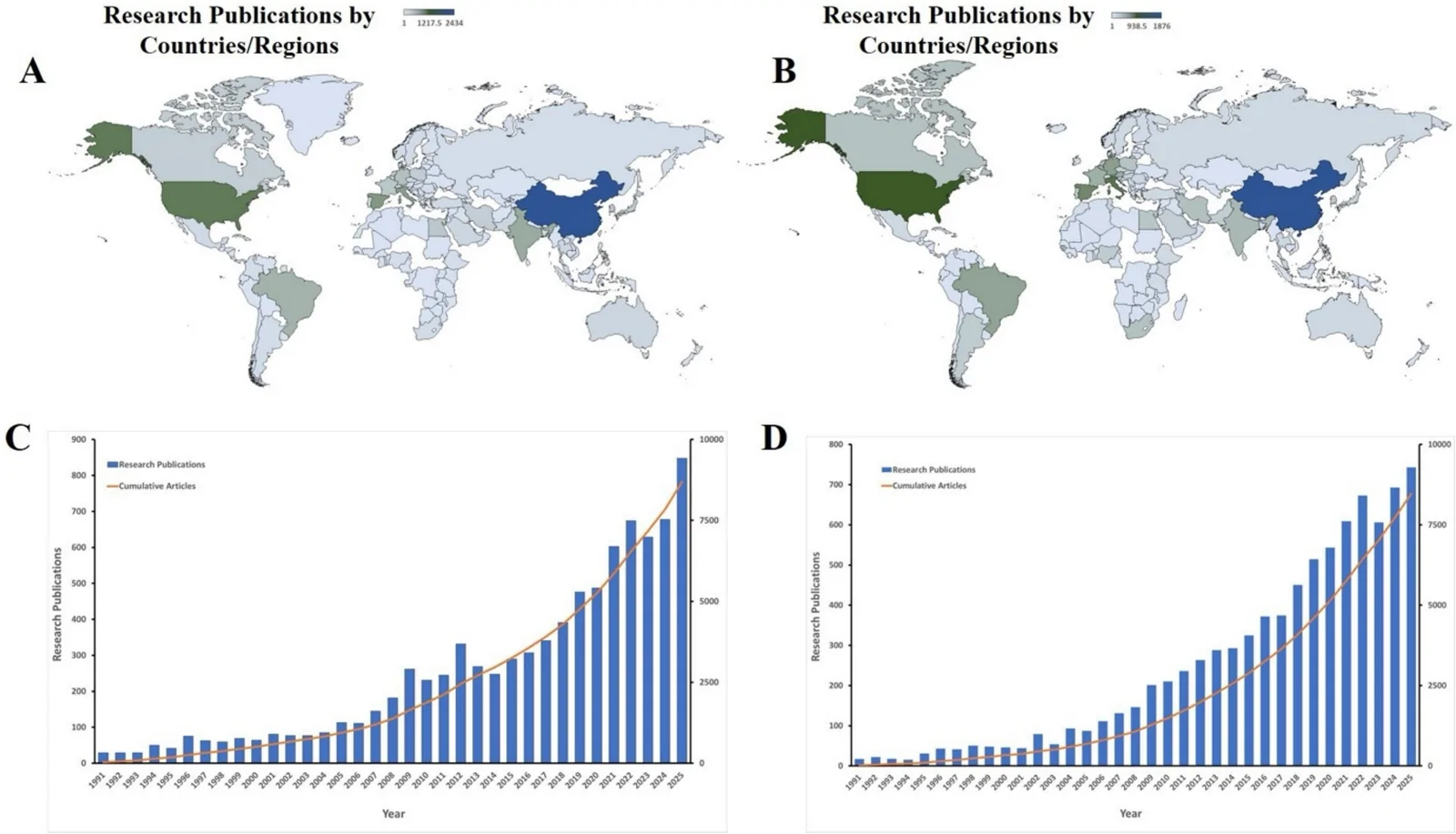
Choropleths maps showing the number of research publications around the globe related to food and pesticide residues (A) and food and mycotoxins (B). The annual number of research publications and the cumulative number of articles indexed in WOSCC related to food and pesticide residues (C) and food and mycotoxins (D) from 01/01/1991 to 31/12/2025.

Conventional methods used for pesticide and mycotoxin detection and monitoring based on chromatographic or spectroscopic techniques are very accurate and sensitive but have limitations such as being time-consuming, requirement of toxic organic solvents, needing trained manpower, being inappropriate for on-site monitoring, requiring expensive reagents and sophisticated equipment, and tedious sample pretreatment and cleanup processes (Jara et al., 2022; Thenuwara et al., 2025). Hence, the need for alternative rapid, easy-to-use, and inexpensive techniques has received considerable attention over the years (Jara et al., 2022).

Proteins are one of the most important biological macromolecules that carry out a wide range of biological activities to enhance the host's fitness and survival (Morris et al., 2022). Proteins contain small subunits called domains, which are involved in functions like catalytic activity, binding with other proteins or biomolecules, signaling, regulation of cellular and metabolic pathways, immunity, and cellular transportation (Morris et al., 2022). In biosensors and other bioanalytical techniques, the two protein domains possessing binding functions (such as antibodies) and catalytic functions (enzymes) are of utmost importance (Kumar et al., 2025). Antibody immobilization is an important process in developing biosensors and other diagnostic tools Proper immobilization ensures stable attachment of the antibody to the transducer surface and preserves the antibody's function and specificity, which are needed for analyte recognition (Kumar et al., 2024).

Bioreceptors used in sensing platforms can be engineered to improve compatibility with the target analyte and enhance the sensor's overall analytical performance (Dkhar et al., 2022). Owing to their high specificity and selectivity, antibodies are commonly employed as bioreceptors in biosensing platforms. The integration of antibodies into various biosensing platforms has significantly advanced the development of both in vitro and in vivo biosensors (Dkhar et al., 2022). Previous reviews on this theme focus heavily on specific antibody-based sensing methods such as immunosensors, lateral flow immunoassays (LFIA), and enzyme-linked immunosorbent assays (ELISA), with a primary focus on classification and working principles. In contrast, this review does not focus on classification; it primarily emphasizes the application of antibody-based sensing technologies for detecting pesticide residues and mycotoxins in fruits, vegetables, cereals, and cereal-based products. In addition, most previously published reviews have focused on a single detection approach, such as biosensors or immunosensors, or on a particular technique for detecting specific contaminants, such as pesticides or mycotoxins. In contrast, this review provides a comprehensive overview of ELISA, LFIA, and immunosensors for the detection of both mycotoxins and pesticides in cereals, fruits, and vegetables (Balkrishna et al., 2024; Ermolaeva et al., 2019; Gai et al., 2023; Ghosh et al., 2022; Maphaisa et al., 2025; Patel, 2025; Reynoso et al., 2019). The review begins by discussing the quality and safety concerns associated with fruits, vegetables, cereals, and cereal-based products. It then delineates various traditional and modern methods used to detect pesticides and mycotoxins in different food matrices. The review provides a comprehensive overview of various antibody-based sensing platforms used to monitor pesticides and mycotoxins. Finally, it discusses the limitations of antibody-based sensing methods and potential strategies to improve the specificity and sensitivity of these devices for detecting mycotoxins and pesticides.

## 2 Methodology

The authors searched academic literature across multiple electronic databases such as Google Scholar, EBSCO, Scopus, PubMed, and Web of Science using a specific set of search terms/keywords. The terms include “pesticides”, “mycotoxins”, “fruits”, “vegetables”, “cereals”, “cereal-based products”, “ELISA”, “LFIA”, “immunosensors”, and “challenges”. The following criteria were established for the inclusion of articles: (1) publication in English and (2) rigorous adherence to an experimental study design that falls between the years 2011 and 2026 and total 67 studies were included.

## 3 Global concern over pesticide residues and mycotoxins

Fig. 2 demonstrates various pesticides and mycotoxins and their detrimental effects on the health of consumers.

**Fig. 2:**
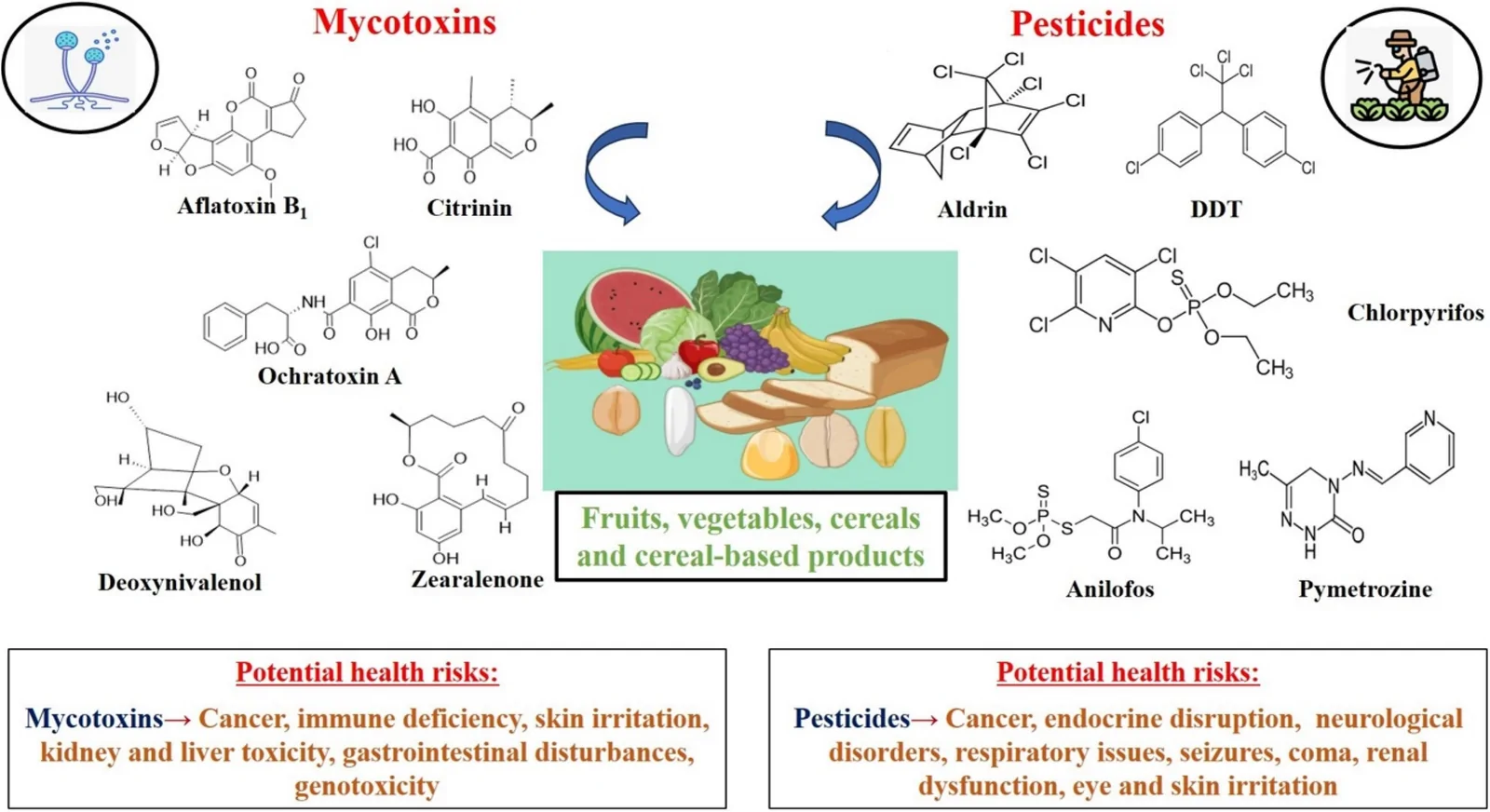
Major pesticide residues and mycotoxins affecting the quality of fruits, vegetables, and cereals and cereal-based products and their negative impact on human health.

### 3.1 Cereals and cereal-based products

#### 3.1.1 Pesticide residues

Various pests and weeds have been continuously affecting the yield and quality of cereal crops for many years. Hence, farmers use different types of pesticides to protect the crops and improve the yield. However, the damage caused by pests continues to be a major problem for farmers despite pesticide use, leading to increased and repeated pesticide use. These activities cause pesticide residues to accumulate in soil and water from where they can reach the food we consume and pose risks to human and animal health (Mishra et al., 2025). Consuming contaminated food crops is one of the main ways humans are exposed to harmful pesticides. Hence, regulatory authorities throughout the globe have established limits for pesticide residues in food products, known as maximum residue levels (MRLs), to protect the consumer health and minimize health risks (Table 1) (Tourinho et al., 2025). MRL can be defined as the highest concentration of pesticide residues permissible in food and animal feed available in the market. MRLs are determined in accordance with good agricultural practices (GAP) and vary across countries because of different socio-demographic factors and government policies (Adhikari & Thapa, 2023). Therefore, the MRL for a specific pesticide can vary in different countries for the same food item.

**Table 1:** Maximum residue levels (MRLs) for certain mycotoxins and pesticides in plant-derived foods set by European Union (EU). **Source:**EUR-Lex, 2023; Kuchheuser & Birringer, 2022

Type of mycotoxin	MRLs	Type of food
Aflatoxin B1	2 μg/kg; 5 μg/kg	Cereals and cereal based products; Maize and rice
Ochratoxin A	5 μg/kg; 3 μg/kg	Unprocessed cereal grains; Products derived from unprocessed cereal grains and cereals placed on the market for the final consume
Patulin	25 μg/kg	Solid apple products
Deoxynivalenol	1250 μg/kg; 1750 μg/kg; 750 μg/kg; 750 μg/kg; 500 μg/kg	Unprocessed cereal grains; Unprocessed maize grains; Cereal flour, semolina, bran and germ as final product; Pasta; Bread, pastries, biscuits, cereal snacks and breakfast cereals
Zearalenone	100 μg/kg; 350 μg/kg; 75 μg/kg; 50 μg/kg	Unprocessed cereal grain; Unprocessed maize grains; Cereals placed on the market for the final consumer, cereal flour, semolina, bran and germ as final product placed on the market for the final consumer; Bread, pastries, biscuits, cereal snacks and breakfast cereals
Fumonisins	4000 μg/kg; 100 μg/kg; 800 μg/kg; 200 μg/kg	Unprocessed maize grains; Maize placed on the market for the final consumer; Maize-based breakfast cereals and maize-based snacks; Baby food containing maize and processed maize-based food for infants and young children
Citrinin	100 μg/kg	Food supplements based on fermented rice
Ergot alkaloids	50 μg/kg	Milling products of barley, wheat, spelt and oats
Type of pesticide	MRLs	Type of food
Chlorpyrifos	0.01 mg/kg	Sweet peppers/bell peppers
Dimethoate	0.01 mg/kg; 0.01 mg/kg	Beans with or without pods; Apples
Carbendazim	0.1 mg/kg; 0.01 mg/kg	Sweet peppers/bell peppers; Rice
Methomyl	0.04 mg/kg; 0.01 mg/kg; 0.1 mg/kg	Sweet peppers/bell peppers; Grapes; Beans with or without pods
Acetamiprid	0.2 mg/kg	Okra/lady's fingers
Omethoate	0.01 mg/kg; 0.01 mg/kg	Eggplants; Apples
Triazophos	0.01 mg/kg	Okra/lady's fingers

It has been estimated that every year about 2 million tonnes of pesticides is used worldwide. Among different pesticides found in food grains, chlorpyrifos is the most common pesticide residue detected in wheat and rice. Other pesticides, such as profenofos, malathion, triazofos, cypermethrin, and lambda-cyhalothrin, are also detected in these crops (Mishra et al., 2025; Sharma et al., 2019). A study by Soman et al. (2024) reported that chlorpropham and chlorpyrifos are common pesticides detected in wheat and rice, with detection rates of 44% and 72%, respectively. Likewise, Mishra et al. (2025) also investigated pesticide residues in rice and wheat grain samples procured from local markets in Haryana, India, using gas chromatography-tandem mass spectrometry (GC–MS/MS). The results revealed the presence of different pesticides like beta-cyfluthrin, buprofezin, chlorpyrifos, hexaconazole, isoprothiolane, and propiconazole in rice. Moreover, the concentration of these pesticides was higher than the permissible limit set by the Food Safety and Standards Authority of India (FSSAI). In contrast, pesticide residue levels in wheat samples were lower than the permissible limit. Further, malathion and chlorpyrifos were predominantly found in wheat, whereas chlorpyrifos and propiconazole were found in rice (Mishra et al., 2025). A similar study by Hakme et al. (2024) analyzed different pesticide residues in barley, oats, rye, and wheat, as well as their processed products such as bran, feed bran, and flour. The authors observed that pesticide residues were present at the highest concentration in bran, then in feed bran, and that the lowest amount of the pesticides was detected in white flour. Furthermore, the analysis of the processing factor showed that the tested pesticide residues were highly concentrated in the wheat and rye bran compared to barley and oats. Moreover, wheat and rye flours had lower pesticide-residue concentrations than the barley and oats flour (Hakme et al., 2024). Another research group studied the presence of 37 pesticides in 209 cereal samples collected from farms, markets, warehouses, and mills in Italy between 2018 and 2019. The study reported that 18 cereal samples had at least one pesticide residue above the quantification limit. However, the level of the pesticides detected in the cereals did not exceed the MRL determined by the European regulatory body (Nardelli et al., 2021). Lozowicka et al. (2014) also analyzed various pesticide residues in barley, rye, oats, and wheat samples collected from local farmers in Kazakhstan. They reported the presence of 10 pesticide residues in cereal grains, including residues of banned pesticides such as aldrin, diazinon, dichlorodiphenyltrichloroethane, and γ-hexachlorocyclohexane. Among the 180 tested pesticides, pirimiphos methyl and chlorpyrifos methyl residues were the most frequently detected in the tested cereals; wheat had the most pesticide-contaminated samples, and rye the fewest (Lozowicka et al., 2014).

#### 3.1.2 Mycotoxins

Mycotoxins, or fungal toxins, are toxic secondary metabolites having a low molecular weight of ∼700 Da secreted by fungi belonging to the *Alternaria, Aspergillus*, *Claviceps, Fusarium*, *Penicillium*, and *Stachybotrys* genera (Kushwaha et al., 2025). Currently known mycotoxins are aflatoxins (AFs), alternariol (AOH), alternariol monomethyl ether, deoxynivalenol, fumonisins (FBs), ochratoxin A (OTA), patulin (PT), sterigmatocystin (STC), tenuazonic acid, trichothecenes (TCTs), and zearalenone (Pandey et al., 2023). These toxins, produced by fungi colonizing various cereals, nuts, fruits, and vegetables, are considered food contaminants because of their detrimental effects on human and animal health. It has been estimated by FAO that nearly 25% of food crops are contaminated with mycotoxins, causing the loss of nearly one billion tons of food and feed products every year (Niaz et al., 2025). However, it has been reported by Eskola et al. (2020) that mycotoxin contamination of global food crops can be in the range of 60–80%, which is much higher than the earlier 25% estimate provided by the FAO. In 2018, wet weather conditions increased mycotoxin contamination, affecting nearly 63.5 million metric tons (Mt), or 2.5 billion bushels, of maize in the USA and Ontario province of Canada (Mueller et al., 2020). In a study by Yilmaz Eker et al. (2023) 140 cereal-based food items like breakfast cereals, cornmeal, maize-based products, and oatmeal sold in the Turkish market were assessed for detecting multiple types of mycotoxins such as AF B1, AF B2, AF G1, AF G2, citrinin (CIT), deoxynivalenol, FB B1, FB B2, HT-2, OTA, T-2, and zearalenone. In that study, it was reported that tested samples had AFB1 (28.57%) and total aflatoxins (B1 + B2 + G1 + G2) (26.43%) concentrations above the level authorized by the Turkish Food Codex. Additionally, mycotoxin contamination above the detection limit was observed in 22.86%–99.29% of the tested cereal-based food items. Furthermore, co-contamination of the tested foods with different types of mycotoxins was also detected in a significant proportion of the samples (Yilmaz Eker et al., 2023). Climate change increased mycotoxin contamination in cereals in southeastern China between 2009 and 2015 (Zhang et al., 2023). Increased rainfall in Jiangsu Province during 2010–2012 caused a spike in deoxynivalenol contamination in wheat. Similarly, maize samples collected in Shanghai showed increased deoxynivalenol contamination during 2009–2012, reporting an average concentration of 130 μg/kg (Zhang et al., 2023). In another study, Dropa et al. (2021) inspected 20 oat flake products obtained from the retail market in the Czech Republic and detected 16 different mycotoxins, among which trichothecenes and enniatins were most predominant, at concentrations ranging from 1 to 156 μg/kg. Moreover, two oat flake samples were reported to contain ochratoxin A levels above the maximum limits prescribed by European Union (EU) regulations (Dropa et al., 2021). Rai et al. (2018) studied zearalenone contamination in 117 cereal samples, comprising corn, rice, wheat, and oats, collected from a local market in India. They reported that 70 of the samples showed zearalenone contamination, and one-third of the contaminated cereals exceeded the maximum permissible limits established by the EU (Rai et al., 2018). A study reported extensive contamination of AF B_1_, OTA, and ZEN in cereal products sold in retail markets in Punjab, Pakistan, based on HPLC analysis of 229 samples. Among the analyzed products, 130 tested positive for zearalenone, 121 were contaminated with AFB1, and 115 tested positive for OTA; many of the samples exceeded the maximum limits for OTA and zearalenone. Furthermore, semolina (Suji) had the highest average concentration of zearalenone, 21.45 ± 1.90 μg/kg (Alim et al., 2018).

### 3.2 Fruits and vegetables

#### 3.2.1 Pesticide residues

FAO estimated that 3.73 million tonnes of pesticides were used in agricultural activities globally in 2023, of which herbicides constituted 51%, followed by fungicides and bactericides (22%), insecticides (22%), and other pesticides accounted for the remaining 5% (FAO, 2025). The top 10 pesticide-consuming countries in 2023 were Brazil (800,652 t), USA (429,501 t), Indonesia (294,935 t), Argentina (262,507 t), China (229,026 t), Australia (182,264 t), Vietnam (161,908 t), Russian Federation (97,018 t), Canada (95,645 t), and France (65,415 t), whereas India ranked 17th (40,094 t) on the list (FAO, 2025). Various international and national regulatory agencies allow the use of pesticides in agricultural activities within specified limits. However, excessive application of various types of pesticides to fruits and vegetables can have detrimental effects on human health as well as on the environment (Balkrishna et al., 2024). Organophosphate pesticides like chlorpyrifos, diazinon, and malathion can cause long-term neurological and psychiatric effects, mainly by inhibiting the acetylcholinesterase enzyme (Naughton & Terry, 2018). Exposure to paraquat can cause severe skin burns, pulmonary fibrosis, and respiratory failure (Ahmad et al., 2024; Zhou et al., 2013). Similarly, carbamate pesticides like sevin, ziram, and carbofuran can cause endocrine disruption, reproductive damage, and increase the risk of non-Hodgkin's lymphoma (Nicolopoulou-Stamati et al., 2016). Several pesticides have been reported to be carcinogenic, and have the ability to cause birth defects, endocrine disruption, neurological damage, and injury to vital organs such as the kidney and liver (Thompson et al., 2017; Thompson & Darwish, 2019). Therefore, it is necessary to monitor pesticides and their residues in various food products regularly. Organic farming avoids the use of chemical pesticides; however, food can still become contaminated through contaminated groundwater and soil, spray drift, or mislabeling conventionally produced food as organic. Therefore, monitoring pesticide residues in conventionally grown as well as in organic food products is necessary to maintain food safety and quality (Balkrishna et al., 2024).

The Rapid Alert System for Food and Feed (RASFF), established in 1979, plays an important role in monitoring food safety in the European Union by alerting when a public health risk is noticed in the food chain. Pesticide residues are the third most reported food safety hazard category in the RASFF database, and between 1981 and 2020, most pesticide residue notifications (67.6%) were related to fruits and vegetables (Pigłowski, 2022). A study conducted by Bakırcı et al. (2014) in the Aegean region of Turkey between 2010 and 2012 inspected 1423 fresh fruit and vegetable samples for 186 pesticide residues. It was reported that 754 samples contained pesticide residues within the limits permitted by Turkish regulations. However, 9.8% of vegetable and 8.4% of the fruit samples exceeded the MRL determined by the Turkish authorities (Bakırcı et al., 2014). Another similar study was conducted in the same region of Turkey between 2012 and 2016 to evaluate the pesticide residues in vegetables and fruits. The study analyzed 3044 fruit and vegetable samples and found that 11.6% of the samples had MRL values higher than the limit established by the Turkish regulatory body (Kazar Soydan et al., 2021). An analysis of 250 vegetable samples collected from farmers' fields in India reported that 34% of samples showed the presence of pesticide residues, and 5.2% of the total samples had residues more than the prescribed MRL (Swarnam & Velmurugan, 2013). Similar studies have been conducted on fruits and vegetables originating from Egypt and Thailand as well, where 16.5% of 176 vegetable samples tested in Egypt in 2018 and 9% of the total 575 samples originating from Thailand in 2011 had pesticide residues above the prescribed MRL (Shalaby et al., 2021; Skretteberg et al., 2015). The EU Multiannual Coordinated Control Programme analyzed 11,727 food samples for pesticide residues and reported that 1.6% of the samples exceeded the MRLs. The programme also reported that the presence of multiple pesticide residues were found in one-third of the analyzed samples, in which fruits and vegetables such as apples (18.6%), strawberries (17.5%), peaches (16.9%), tomatoes (14.2%) and lettuces (12.4%) were the food items having the highest frequency of multiple residues presence (EFSA et al., 2024). Furthermore, analysis of RASFF data from 1999 to 2022 by Eissa et al. (2024) showed extensive contamination of vegetables and fruits with multiple pesticide residues. Fruits and vegetables accounted for 67.1% of all pesticide residue notifications reported in the RASFF database, with sweet peppers having the most notifications related to pesticide residues. Okra, chili peppers, beans, and aubergines had the highest rates of pesticide contamination: 39.69%, 37.07%, 32.46%, and 30.53%, respectively. Among the fruit samples, apples (27.17%), pomegranates (26.13%), and strawberries (22.92%) had the highest contamination rates, and pears and tomatoes showed the lowest contamination of pesticides, i.e., 5.69% and 6.93%, respectively (Eissa et al., 2024).

#### 3.2.2 Mycotoxins

A plethora of studies have reported mycotoxin contamination in fruits. Grapes have been found to contain mycotoxin-producing fungi. The mycotoxin-producing fungi constituted 8.0% of the total grape mycoflora, where potential producers of ochratoxin A were the most prevalent (6.0%), followed by trichothecene-producing fungi (1.2%), patulin-producing fungi (0.5%), and aflatoxin-producing fungi (0.3%) (Serra et al., 2005). A study analyzed dried vine fruits, sultanas, raisins, and currants, obtained from a retail market in the United Kingdom for the presence of ochratoxin A. The study reported that ochratoxin A was detected in 88% of the samples, with contamination above 0.2 μg/kg present in (17 of 20) sultana, (17 of 20) raisin, and (19 out of 20) currant samples (MacDonald et al., 1999). In Pakistan, 21.8% (29 out of 133) mango or mango-based products and 0.7% (1 out of 141) orange or orange-based products were reported to have patulin level more than the regulatory limit of 50 μg/kg (Hussain et al., 2020). Furthermore, the study reported that mango fruits were more prone to patulin contamination than mango juice, mango pulp, or oranges (Hussain et al., 2020). Similarly, another study from Pakistan detected high patulin concentrations ranging from 0.04 to 1100 μg/kg in fruits and fruit juices, where red globe grape samples had the highest average level of patulin (921.1 ± 22.4 μg/kg). Furthermore, the study reported that 33.8% of the samples had patulin levels higher than the MRL of 50 μg/kg prescribed by the EU (Iqbal et al., 2018). A large-scale study conducted in Argentina between 2005 and 2013 reported the detection of patulin and ochratoxin A in different fruits and fruit products such as juices and pulps. Patulin was detected in 1997 of 5958 samples at concentrations ranging from 3.0 to 19,622 μg/L. Furthermore, the study reported that apples had the highest percentage of patulin-contaminated samples, and they also had the highest patulin concentration of 19,622 μg/L among all the fruit commodities. In the case of ochratoxin A, 22 out of 1401 samples tested positive for the mycotoxin, where grapes accounted for the maximum number of ochratoxin A contaminated samples and also exhibited the highest ochratoxin A concentration of 3.6 μg/L (Oteiza et al., 2017).

## 4 Conventional techniques used for determining the safety and quality of vegetables, fruits and cereals

A significant amount of research has been conducted over the years to develop screening and monitoring technologies for detecting pesticides and mycotoxins in various fruits, vegetables, cereals, and cereal-based products. This has led to the development of many analytical instruments based on chromatographic and spectroscopic techniques that aim to achieve the detection of the adulterants in a sensitive, specific, quick, and cost-effective way, with the capacity for on-site detection (Ghosh et al., 2022; Maphaisa et al., 2025). Despite technological advancements so far, the majority of the currently available adulterant detection methods for various food items are incapable of offering all of these analytical properties (Table 2).

**Table 2:** Comparative analysis of various routine techniques to detect the pesticide residues and mycotoxins in foods.

Technique	Advantages	Limitations
Thin layer chromatography (TLC)	➢ Easy to use with minimal training ➢ Allows for more considerable sample analysis as a first screening method at low cost ➢ Requires less sample preparation	➢ Limiting separation time ➢ Labor-intensive ➢ Low reproducibility and resolution
High-performance liquid chromatography (HPLC)	➢ High separation efficiency ➢ Good selectivity ➢ Extremely quick ➢ High detection sensitivity	➢ Costly ➢ Complex ➢ Requires large quantities of expensive organic compounds
Gas chromatography-flame photometric detector (GC-FPD)	➢ Highly sensitive detection ➢ Quick ➢ Easy ➢ Cheap ➢ Effective ➢ Rugged ➢ Safe	➢ Filter must be exchanged between chromatographic runs
Gas chromatography-mass spectrometry (GC–MS)	➢ Very good recovery value ➢ Effective ➢ Use of analyte protectants ➢ Sensitive ➢ Selective	➢ Clean-up performance is not good enough ➢ When analyzing complex samples, it is common that chromatography cannot completely separate some com ponents and may generate overlapped peaks ➢ The presence of co-eluting components may hinder both the identification and quantification ➢ Noise, baseline and retention time shift further increase the difficulty to extract chemical information
Liquid chromatography-mass spectrometry (LC-MS)	➢ Effective ➢ Rugged ➢ Safe ➢ Adaptable ➢ Selective ➢ Simple, does not require a mass of toxic organic solvents ➢ Allows processing a significantly larger number of samples in a short time ➢ Identify solutes in low concentrations (which are in parts per million (ppm)) in a complex mixture	➢ Higher operational cost ➢ More limited sample throughput and less favourable ➢ Only works with volatile buffers that are required to avoid fouling of the atmospheric pressure ionization (API) interface ➢ Residual impurities being analyzed should be ionized
Near-infrared spectroscopy (NIR)	➢ Quick and automatic ➢ Easy to use ➢ High penetration depth ➢ Reproducible	➢ Low sensitivity ➢ High cost

**Source:**Maphaisa et al., 2025 an open access article under the CC BY license; Fan et al., 2022; Ghosh et al., 2022 under a Creative Commons Attribution 4.0 International License.

## 5 Role of ELISA in food safety and quality

ELISA could be a promising alternative to the conventional tools for detecting pesticides and mycotoxins in food samples. ELISA utilizes antigen-antibody specificity to rapidly identify a wide variety of adulterants with minimal equipment and resources, in a simple, cost-effective manner with high specificity (Asensio et al., 2008; Du et al., 2023). There are different types of ELISA, such as direct, indirect, sandwich, and competitive; among these, indirect and sandwich ELISA are commonly used for food adulterant detection (Asensio et al., 2008). The following sections discuss the use of different ELISA types in ensuring food safety and quality.

### 5.1 Cereals and cereal-based products

#### 5.1.1 Pesticide residues

Yin, Li, et al. (2023) have developed an indirect competitive ELISA (icELISA) for detecting the presence of the neonicotinoid insecticide pymetrozine in different types of food samples by using anti-pymetrozine monoclonal antibodies. The icELISA was reported to be specific to pymetrozine and did not show cross-reactivity with other pesticides such as nitenpyram, metanalyzin, metamitron, imidacloprid, hexazinone, and acetamiprid that have similar structures. Furthermore, the icELISA had an LOD ranging between 1.62 and 1.66 μg/kg in cereals, namely wheat, maize, and rice. Additionally, study reported that the sensitivity of icELISA in detecting pymetrozine was in line with the sensitivity of LC-MS/MS. Additionally, the icELISA has other advantages like low cost, less analysis time and ease of operation than LC-MS/MS (Yin, Zhang, et al., 2023). In another study, a direct competitive ELISA (dcELISA) was designed to detect metsulfuron-methyl herbicide residues in oat, wheat, and rice samples. The study reported that the dcELISA was highly sensitive, having an IC_50_ value of 37.03 μg/L. Furthermore, dcELISA had recovery rates ranging from 81.15% to 101.85% in the spiked rice, wheat, and oat samples. Moreover, the ELISA had a detection limit of 0.01 mg/kg in the tested cereal samples, and the results were comparable to those obtained by HPLC-MS (Liu, Qin, et al., 2021). Similarly, another study used dcELISA to detect another herbicide, anilofos, in cereals such as wheat, corn, barley, and different varieties of rice. The dcELISA detected anilofos without cross-reactivity and had an LOD of 2 μg/kg in the tested barley, wheat, brown rice, white rice, and aromatic rice samples (Zhang et al., 2013). An ELISA using carbaryl-specific camelid heavy-chain antibody variable domains (VHH) generated from an immunized alpaca has been developed for the detection of the insecticide carbaryl in maize, rice, and wheat samples. The VHH-based ELISA showed low cross-reactivity (≤0.8%) toward other carbamate pesticides, and the detection limits of the insecticide in wheat were 72 ng/g and 36 ng/g for maize and rice samples (Liu et al., 2019). The residues of the fungicide picoxystrobin in barley, corn, oat, and wheat flour samples were detected using a competitive enzyme-linked immunosorbent assay, which had an LOD of ≤0.1 μg/L and showed recovery rates ranging between 84 and 115% (Mercader et al., 2012).

#### 5.1.2 Mycotoxins

Sekizuka et al. (2025) developed a dcELISA for the detection of the trichothecene mycotoxin nivalenol in contaminated wheat and barley. The findings revealed that dcELISA has a linear detection range between 0.62 and 13.2 ng/mL for nivalenol. Moreover, other mycotoxins like deoxynivalenol and 4-acetyl nivalenol did not present any cross-reactivity within the assay. Further, recovery percentages of spiked samples of barley and wheat were determined to be 80–84% and 76–81%, respectively (Sekizuka et al., 2025). Similarly, Boshra et al. (2023) used a commercial ELISA kit to detect the mycotoxins, namely aflatoxins, deoxynivalenol, ochratoxin A, and zearalenone in cereals such as oat, rice, and wheat as well as in cereal-based products like pasta and noodles sold in the Egyptian markets (Boshra et al., 2023). Analysis of 70 cereal samples reported that 3.9% of locally sourced samples had ochratoxin A levels above the permitted limit approved by the Egyptian regulatory authority. Similarly, 17.6% of imported pasta and noodle samples exceeded the maximum permissible limit for deoxynivalenol (>750 μg/kg). However, the tested cereal samples were free from deoxynivalenol, zearalenone, and aflatoxin contamination (Boshra et al., 2023). In another study, commercially available ELISA kits were also used to determine the levels of aflatoxin B1, deoxynivalenol, fumonisins, ochratoxin A, and zearalenone in unprocessed cereals and cereal-based products grown either organically or conventionally in Croatia. It was reported that the detected mycotoxin levels in the tested samples, which included Barley, maize, maize flour, maize meal, oat, oat mash, rye, wheat, and wheat flour, posed no significant health risk to the consumers (Pleadin et al., 2017). A sandwich dot-ELISA (s-dot ELISA) has also been developed for the detection of ochratoxin A in maize, rice, and wheat samples collected from storage facilities and agricultural fields in Karnataka, India. Out of the 195 cereal samples, 72 were found to be contaminated with ochratoxin A, and the s-dot ELISA was reported to have high specificity for OTA without any cross-reactivity toward other mycotoxins such as aflatoxin B1, deoxynivalenol, and fumonisin B1 (Venkataramana et al., 2015). Table 3 lists the various ELISA developed for mycotoxin detection in various cereal and cereal-based foods.

**Table 3:** Detection of mycotoxins in cereals and cereal-based products using an in-house developed enzyme-linked immunosorbent assay (ELISA).

Country of study	Type of ELISA	Type of cereals/cereal-based products	Targeted mycotoxin	Detection limit	References
China	Indirect competitive	Maize (spiked)	Aflatoxin B1	6.58 ng/kg (matrix based)	Han et al., 2024
China	Indirect competitive	Wheat (spiked), and corn (spiked)	Ochratoxin A	0.0015 ng/mL (solvent based)	Ren et al., 2023
Netherlands	Competitive	Rice (spiked), wheat (spiked), and maize (spiked)	Sterigmatocystin	1.5 μg/kg (matrix based)	Oplatowska-Stachowiak et al., 2018
China	Indirect competitive	Red yeast rice (spiked)	Citrinin	0.01 ng/mL (solvent based)	Kalayu Yirga et al., 2017
Finland	Noncompetitive	Wheat (spiked), barley (spiked), and oat (spiked)	HT-2 toxin	0.3 ng/mL (matrix based)	Arola et al., 2017
China	Indirect competitive	Corn (spiked)	Fumonisin B1	1.15 ng/mL (solvent based)	Wang et al., 2016
China	Indirect competitive	Maize (spiked), and wheat (spiked)	Deoxynivalenol	6.12 ng/mL (solvent based)	Li et al., 2012
Italy	Direct competitive	Wheat flour (real sample)	Deoxynivalenol, and ochratoxin A	550 μg/kg for deoxynivalenol, and 1.8 μg/kg for ochratoxin A (matrix based)	Folloni et al., 2011

### 5.2 Fruits and vegetables (F & V)

#### 5.2.1 Pesticide residues

An indirect competitive ELISA (icELISA) has been developed for the detection of fungicide pyrimethanil residues in fruits and vegetables such as apple, cucumber, grape, peach, strawberry, and tomato. The assay had an LOD ranging from 0.02 to 0.04 μg/kg and recovery rates between 88.6 and 102.3% (Cao et al., 2019). Similarly, in another study, a chemiluminescent dcELISA was developed for detecting the herbicide 2,4-dichlorophenoxyacetic acid (2,4-D), which showed higher levels of 2,4-D in peels of oranges (79–104 μg/kg) compared to mandarin peels obtained from the fruits sold in markets of Vietnam (1.66–2.82 μg/kg) (Vdovenko et al., 2013). Hua et al. (2012) developed icELISA for detecting fenitrothion, parathion, and parathion-methyl organophosphorus pesticide residues in spiked samples of apple, Chinese cabbage, and green grocery (Hua et al., 2012) (Table 4).

**Table 4:** Detection of pesticide residues in fruits and vegetables (F & V) using an in-house developed enzyme-linked immunosorbent assay (ELISA).

Country of study	Type of ELISA	Type of F & V	Targeted pesticide	Detection limit	References
China	Indirect competitive	Broccoli, and cabbage (spiked)	Pymetrozine	2.67 μg/kg for broccoli, and 2.50 μg/kg for cabbage	Yin, Li, et al., 2023
Thailand	Indirect competitive	Coriander, yard long bean, pakchoi, cabbage, Chinese cabbage, morning glory, cauliflower, spring onion, broccoli, chili, Thai eggplant, Chinese kale, cucumber, tomato	Chlorpyrifos	38.3 μg/kg for coriander; 16.6 μg/kg for yard long bean; 30.8 μg/kg for pakchoi; 13.4 μg/kg for cabbage; 332 μg/kg for Chinese cabbage; 32.8 μg/kg for morning glory; 16.4 μg/kg for cauliflower; 18.6 μg/kg for spring onion; 9 μg/kg for broccoli; 24.5 μg/kg for chili; 6.20 μg/kg for Thai eggplant; 30 μg/kg for Chinese kale; 146 μg/kg for cucumber; 23.5 μg/kg for tomato (all real samples)	Hongsibsong et al., 2020
China	Indirect competitive	Long bean, chives; mango, and watermelon (all real samples)	Carbofuran and 3-hydroxycarbofuran	0.76 ng/mL for carbofuran, 0.69 ng/mL for 3-hydroxycarbofuran	Lan et al., 2019
China	Indirect competitive	Broccoli, cabbage, tomato, cucumber, celery; tangerine, apple, pear, and banana, papaya (all spiked)	Difenoconazole	4.58 μg/L	Liu et al., 2018
China	Indirect competitive	Cucumber, cabbage, lettuce, tomato; apple, and peach (all spiked)	O,O-diethyl and *O*,*O*-dimethyl (moieties)	NS	Zhao, Dong, et al., 2015

NS: Not specified.

#### 5.2.2 Mycotoxins

Pavón et al. (2012) developed a dcELISA to detect ochratoxin A in dried fig samples. The dcELISA detected ochratoxin A in 19 of 35 fig samples (54.3%) obtained from the Spanish retail market, with concentrations ranging between 3.15 and 277 ng/g, and five samples exceeded the European Commission permissible limit of 10 ng/g prescribed for dried vine fruits (Pavón et al., 2012).

Most studies have employed competitive ELISA (C-ELISA) for the detection of food contaminants. A review written by Xiong et al. (2020) also mentioned that competitive enzyme-linked immunosorbent assay (Cp-ELISA) is the most widely used analytical approach for detecting chemical contaminants in food because of its high throughput, cost-effectiveness, and straightforward readout.

## 6 LFIA in food safety and quality determination

LFIA has emerged as a promising alternative to ELISA because of its properties like short detection time, ease of operation, and low cost (Kim, Shin, et al., 2023). LFIA can be classified into two categories: competitive and direct (non-competitive or sandwich), based on the detection format used (Li et al., 2023). Competitive LFIA is commonly used for the detection of small molecules having limited antigenic sites, such as mycotoxins, pesticides, and residues of veterinary drugs, through competitive inhibitory immunological reactions. In contrast, direct LFIA is mainly employed to detect antigens and antibodies, such as pathogen-associated proteins and human or animal proteins, making it suitable for disease diagnosis and biochemical analysis (Li et al., 2023). From the perspective of sensor design, LFAs are broadly classified into two major formats: competitive assays and sandwich (non-competitive) assays (Pedreira-Rincón et al., 2025). In non-competitive assays, the intensity of the test line (TL) rises with increasing target concentration. In contrast, competitive assays exhibit an inverse relationship, with TL intensity decreasing as the target concentration in the sample increases (Pedreira-Rincón et al., 2025). Immune-sandwich LFAs are used to detect target molecules containing at least two distinct epitopes, which enable the formation of an immuno-sandwich complex involving the antigen and two or more specific antibodies. These assays provide a more intuitive user experience and straightforward result interpretation because the generated signal is directly proportional to the concentration of the target analyte (Pedreira-Rincón et al., 2025). However, competitive LFAs have usually been used for detecting small molecules or targets having single epitopes, where there is difficulty in forming an immune-sandwich complex. Competitive LFAs offer several advantages over the non-competitive ones, like the need for a single bioreceptor and reduced susceptibility to the hook effect. The hook effect occurs when very high analyte concentrations saturate the antigen-binding sites, resulting in a reduced signal and potentially causing false-negative results (Pedreira-Rincón et al., 2025). Research in materials science over the years has helped in the development of a plethora of nanoparticles that can be used in LFIAs to improve the sensitivity of LFIA strips. These nanomaterials possess various unique optical, structural, electronic, and magnetic properties such as strong adsorption ability, good electrical conductivity, biocompatibility, and structural stability, making them suitable for labeling antigens and antibodies (Xue et al., 2019). Some of the nanomaterials that have been applied in LFIA development are carbon nanoparticles (CNPs), fluorescent microspheres, gold nanoparticles (AuNPs), magnetic nanoparticles (MNPs), colloidal selenium nanoparticles (SNPs), and quantum dots (QDs) (Li et al., 2023).

### 6.1 Cereals and cereal-based products

#### 6.1.1 Pesticide residues

Qin et al. (2025) developed a time-resolved fluorescent microspheres-based LFIA (TRFMs-LFIA) (competitive) for the detection of insecticides named clothianidin and dinotefuran in wheat samples. The TRFMs-LFIA has been reported to be very sensitive and specific to the tested insecticides. Moreover, the LFIA did not cross-react with tetrahydrofuran, a structural analogue of clothianidin and dinotefuran. Furthermore, results of TRFMs-LFIA were comparable to UPLC-MS/MS (Qin et al., 2025). Additionally, TRFMs-LFIA (competitive) was also used to detect the fungicide paclobutrazol in both wheat and rice samples. The results revealed an LOD of 1.72 ng/mL for paclobutrazol by TRFMs-LFIA (Cheng et al., 2022). Likewise, Zeng et al. (2025) also developed an LFIA (competitive) for the rapid and quantitative detection of cyclosulfamuron in cereals, with an LOD of 2.18 μg/kg for corn and 1.93 μg/kg for brown and white rice as illustrated in Fig. 3. In another study Huang et al. (2024) developed a gold nanoparticle-based LFIA (Au-LFIA) (competitive) using the 2H1C12 monoclonal antibody for detecting the fungicide tebuconazole residues that had LOD of 35.5 ng/g in wheat samples. Similarly, in a different study, Au-LFIA (competitive) was used for the detection of another fungicide, tricyclazole, in rice samples, where the LOD was 13.58 μg/kg in brown rice and 6.74 μg/kg in polished rice (Liu et al., 2024). Furthermore, the same technology has been employed to detect flutriafol and isoprothiolane in rice samples (Liu, Liu, et al., 2023; Liu, Xu, et al., 2023). The Au-LFIA (competitive) for isoprothiolane detection has an LOD of 66 μg/kg in polished rice and 81 μg/kg in brown rice samples, while the LOD for flutriafol in rice samples was 0.045 mg/kg (Liu, Liu, et al., 2023; Liu, Xu, et al., 2023).

**Fig. 3:**
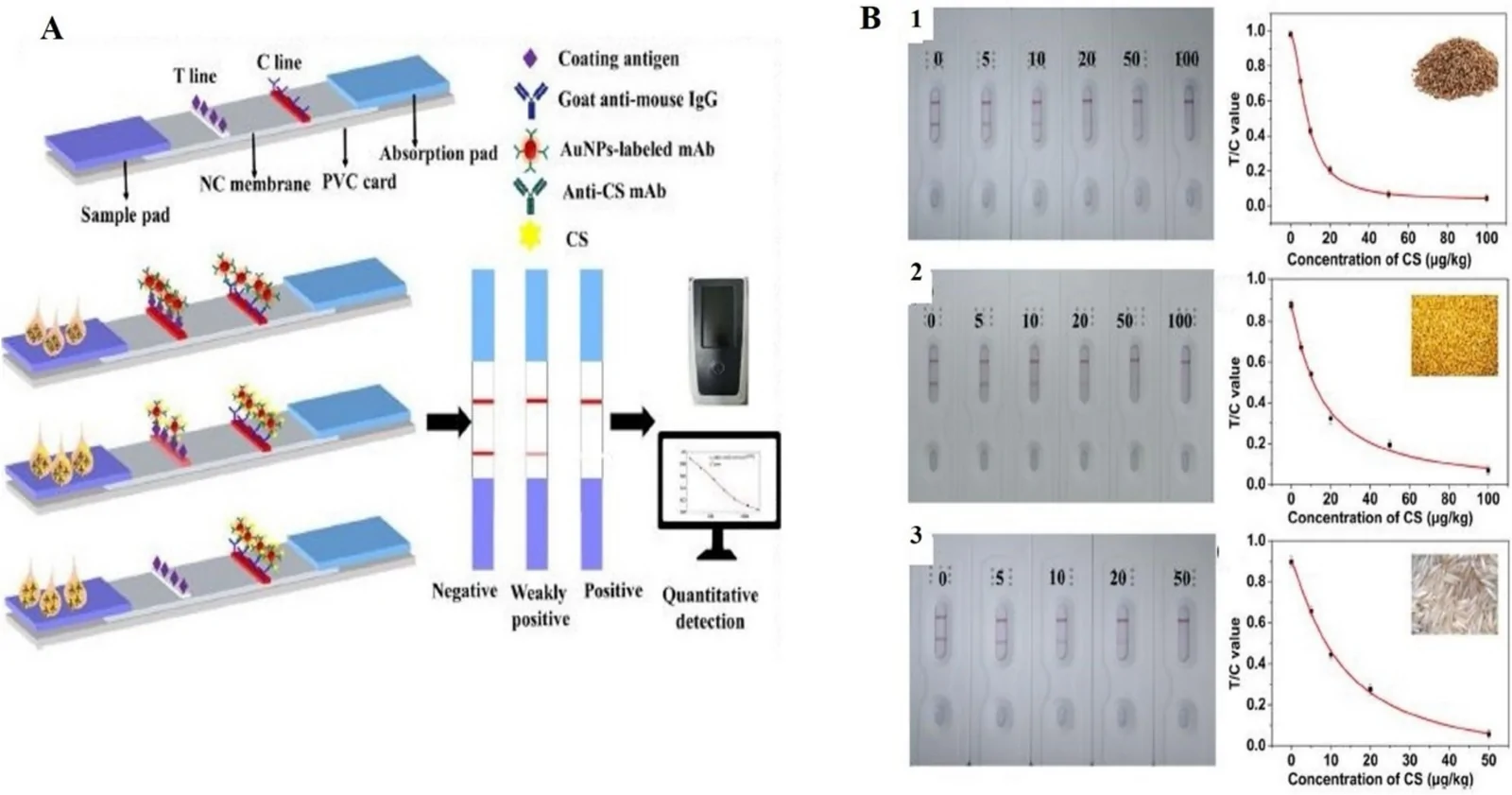
(A) Graphical presentation of LFIA strip detecting the cyclosulfamuron (CS). (B) CS detection in brown rice (B1), corn (B2) and white rice sample (B3) along with their calibration curves (Zeng et al., 2025 with license number 6275921331220). T: test line; C: control line; NC membrane: nitrocellulose membrane; PVC card: polyvinylchloride backing card; T/C value: test-to-control line ratio. (For interpretation of the references to color in this figure legend, the reader is referred to the web version of this article.)

#### 6.1.2 Mycotoxins

A multicolor competitive-LFIA has been developed for the detection of zearalenone and aflatoxin B1 simultaneously using differently colored aggregation-induced emission nanoparticles as signal tags (Zhang et al., 2024). The assay enabled accurate quantitative detection of the mycotoxins, with detection limits of 26 pg/mL for zearalenone and 6.12 pg/mL for aflatoxin B1, and showed good reliability and the ability to detect two mycotoxins in cereal samples such as corn and wheat at once (Zhang et al., 2024). Wu et al. (2023) developed a competitive LFIA using quantum dots nanobead (QBs) to detect aflatoxin B1 and ochratoxin A in corn, rice, and soybean samples. The developed assay showed high sensitivity for both aflatoxin B1 and ochratoxin A mycotoxins with recovery rates within the range of 80.20–130.05%. Additionally, QBs-LFIA was determined to be more sensitive than the traditional LFIA, with no cross-reactivity with deoxynivalenol, fumonisin B1, T-2 toxin, and zearalenone. In another study, a multiplex LFIA (competitive) using amorphous carbon nanoparticles (ACNP-LFIA) as labels was developed for the detection of *Fusarium* mycotoxins in maize samples (Zhang et al., 2017). The ACNP-LFIA had a better visual LOD than gold nanoparticles and quantum dots. Furthermore, the ACNP-LFIA achieved an LOD of 1 μg/kg for zearalenone, 13 μg/Kg for T-2 toxin, and 20 μg/kg for deoxynivalenol, with recovery rates ranging from 84.6% to 109% in spiked maize samples.

### 6.2 Fruits and vegetables (F & V)

#### 6.2.1 Pesticide residues

An AuNP-LFIA and a gold nanoflower-based LFIA (AuNF-LFIA) (competitive) for detecting acifluorfen residues in fruits (tangerine) and vegetables (Chinese cabbage) have been developed by Jiang et al. (2026). The AuNF-LFIA showed improved sensitivity in contrast to AuNP-LFIA, with an LOD of 7.79 ng/mL, i.e., 9.6-fold lower than the LOD of AuNP-LFIA. Furthermore, it was reported that the results generated by both assays were in line with LC-MS analysis (Jiang et al., 2026). Several other studies have also used nanoparticles in LFIA for pesticide residue detection in a plethora of fruits and vegetables. Liu et al. (2025) investigated the use of plasmonic gold nanostars (AuNSs) in the development of novel colorimetric (color-based detection of substances) and photothermal lateral flow immunoassays (LFIAs) (competitive) for the detection of carbendazim in cabbage, chives, and citrus fruit. The results showed an LOD of 0.28 ng/mL for the photothermal LFIA. However, the colorimetric assay was determined to have a higher LOD of 0.48 ng/mL in selected vegetable samples (Table 5). Shi et al. (2024) used the TRFM-LFIA (competitive) for detecting the presence of triadimefon and its residues in oranges, tomatoes, and cucumber samples. It was reported that the TRFM-LFIA attained an LOD of 0.135 μg/kg in cucumbers, 1.047 μg/kg in tomatoes, and 5.811 μg/kg in oranges. Moreover, it provided the results within 18 min without needing complex instrumentation or skilled personnel (Shi et al., 2024). An AuNP-LFIA (competitive) developed by Chen et al. (2023) detected diafenthiuron residues in cabbage and apple samples and had an LOD of 3.4 mg/kg in apples and 1.5 mg/kg in cabbage.

**Table 5:** Detection of pesticide residues in fruits and vegetables (F & V) using an in-house developed lateral flow immunoassay (LFIA).

Country of study	Type of LFIA	Type of nanomaterial used	Type of F & V	Targeted pesticide	Detection limit	References
Thailand	Competitive	Gold nanoparticles (AuNPs)	Banana, apple, grape; tomato, and cabbage	Dichlorvos	44 μg/kg for banana, 93 μg/kg for apple, 16 μg/kg for grape; 19 μg/kg for tomato, 50 μg/kg for cabbage	Jia et al., 2025
Morocco	Competitive	AuNPs	Apple, grape; tomato, onion, and green beans (all real samples)	Carbendazim	1 μg/kg for apple, 2 μg/kg for grape; 9 μg/kg for tomato, 2 μg/kg for onion	Sbihi et al., 2025
China	Competitive	AuNPs	Grape, blueberry; tomato (all spiked)	Flupyradifurone	1 mg/kg	Lu et al., 2024
China	Competitive	AuNPs	Watermelon, and pear (all spiked)	Butralin	10 ng/mL	Kou et al., 2024
China	Competitive	AuNPs	Grape; tomato, and cucumber (all spiked)	Zoxamide	3.44 ng/g for grape; 4.78 ng/g for tomato, 3.53 ng/g for cucumber	Cheng et al., 2023
China	Competitive	AuNPs	Chinese cabbage, lettuce, garlic chives, cucumber and cowpea (all spiked)	Propiconazole	80 ng/g	Xu et al., 2022
China	Competitive	AuNPs	Cucumber; apple (all spiked)	Pyridaben	25 ng/g	Liu, Xu, et al., 2021

#### 6.2.2 Mycotoxins

Cheng et al. (2018) developed an immunochromatographic strip (competitive) that could detect citrinin in fruit samples (ananas, orange, and pear) within 5 min. Furthermore, the strip had a detection limit of 50 ng/mL and did not cross-react with other mycotoxins such as Ochratoxin A, patulin, Fumonisin B1, and Aflatoxin B1. Moreover, it was reported that the immunochromatographic strip results were consistent with HPLC and icELISA when the concentration of citrinin was more than 50 ng/mL (Cheng et al., 2018).

Competitive-LFIA was the most extensively studied LFIA format in the reviewed literature for the detection of pesticides and mycotoxins in cereals, fruits, and vegetables (Table 5). Among the various materials employed for LFIA development, AuNP-based composites were predominantly used in sensor fabrication for the detection of these contaminants. AuNPs are a cost-effective labeling option because they can be synthesized using simple, inexpensive methods, are widely commercially available, and require minimal instrumentation for signal detection, often allowing visual interpretation by the naked eye or through basic colorimetric quantifications. In comparison, alternative labels such as europium nanoparticles, fluorescent dyes, magnetic nanoparticles, and quantum dots may provide higher sensitivity but generally involve higher material costs, more complex fabrication procedures, and less well-established commercial supply chains (Pedreira-Rincón et al., 2025).

## 7 Application of immunosensors in monitoring food safety and quality

The immunosensors can be valuable alternatives to currently available analytical devices because of their properties, such as the possibility of miniaturization and portability, ease of use, and high specificity and sensitivity for the detection of mycotoxins and pesticides in fruit, vegetables, cereals, and cereal-based products (Akbari-Alavijeh et al., 2025; Kumar et al., 2024). Immunosensors are solid-state biosensors that apply antibodies to recognize analytes and couple the immunochemical reaction to a transducer element, which converts the immunochemical reactions to measurable physical signals (Cristea et al., 2015; Van Emon, 2011). Based on the type of transducer used in immunosensors, they can be classified as microgravimetric, thermometric, optical, and electrochemical (Cristea et al., 2015). Lately, with the advancements in nanotechnology, nanomaterial incorporation in immunosensors has been garnering attention. Nanoparticles have a small size, so their chemical and physical characteristics are affected by binding to target molecules, which enables their application in different analytical techniques (Naresh & Lee, 2021). Furthermore, the high surface area-to-volume ratio of nanoparticles can lead to swift detection and provide high sensitivity during the detection of different food contaminants (Wang & Yu, 2013). Additionally, nanomaterials can be used to modify electrodes in electrochemical immunosensors to improve their sensitivity (Martinkova et al., 2017). Furthermore, characteristics of nanomaterials can be customized by modifying their size, shape, composition, and surface functionalization. By controlling the size, composition, and binding interactions of nanomaterials, the sensors' conductive, electronic, spectroscopic, and light-scattering properties can be adjusted for detecting pesticides and mycotoxins (Stephen Inbaraj & Chen, 2016).

### 7.1 Cereals and cereal-based products

#### 7.1.1 Pesticide residues

An electrochemical immunosensor based on a hapten-protein conjugate linked to copper nanoparticles (CuNPs) as electrochemical labels for detecting carbaryl residues in corn, oat, and wheat flours has been designed by Dorozhko et al. (2021). The nanoparticle-based immunosensor had an LOD of 0.08 μg/kg in cereal flours and did not cross-react with carbosulfan and carbofuran, which are other analogues of carbamate pesticides (Dorozhko et al., 2021). In addition, the reproducibility was evaluated by determining the coefficient of variation (CV) for 10 measurements conducted on the same day (intraday) and six measurements performed on different days (interday) using standard carbaryl solutions at different concentrations of 0.08, 6.0, and 32.2 μg/kg. The intraday CV values obtained from ten replicates at each concentration were below 8%, whereas the interday values based on six replicates for each concentration remained below 12% (Dorozhko et al., 2021). Likewise, Boroduleva et al. (2017) fabricated a fluorescence polarization immunoassay (FPIA) to detect the presence of pesticide residues of carbaryl and triazophos in wheat grains. The findings revealed that FPIA has an LOD of 20 μg/kg for carbaryl and 40 μg/kg for triazophos.

#### 7.1.2 Mycotoxins

An electrochemical immunosensor with graphene oxide was developed on screen-printed paper electrodes for detecting aflatoxin B1 in wheat flour with high sensitivity (Chauhan et al., 2025). The graphene oxide-based immunosensor had an LOD of 1.26 fg/μL with high sensitivity (10,186.51 μA/cm^2^/ng) (Chauhan et al., 2025). Pérez-Fernández et al. (2025) developed an electrochemical immunosensor with screen-printed carbon electrodes for detection of total fumonisins (FB1, FB2, and FB3) in maize flour and tortillas. The immunosensor showed high specificity, limits of quantification of 20.25 μg/kg, and an LOD of 6.07 μg/kg in maize samples (Fig. 4) (Pérez-Fernández et al., 2025). The modified electrodes performed well for at least 60 days when kept at 4 °C, maintaining a stable blank-to-signal ratio, thus demonstrating satisfactory stability even after long-term storage (Pérez-Fernández et al., 2025). Again, for the detection of aflatoxin B1 in cereal samples, a smartphone-based magneto-immunosensor using carbon black-modified electrodes was developed by Jafari et al. (2022). The immunosensor was highly sensitive and reproducible, and had good selectivity for aflatoxin B1, with detection limits of 24 pg/mL in corn extract samples. Furthermore, the immunosensor was integrated with the Android application “AflaESense,” which displayed results in a traffic-light format to allow quick and easy detection of mycotoxins in cereal samples. Additionally, AflaESense reported recovery rates of 80–120%, which are within the limits prescribed in the EU guidelines for aflatoxin B1 screening tests (Jafari et al., 2022). Similarly, Valera et al. (2019) developed an electrochemical immunosensor for the detection of deoxynivalenol in wheat samples, which had an LOD of 342.4 μg/kg and a working range between 610 and 6210 μg/kg (Valera et al., 2019). A nanobody (Nb28) mediated Förster resonance energy transfer (FRET)-based immunosensor employing quantum dots has been developed, which can detect ochratoxin A with high selectivity, rapidly (5 min) and had LOD of 0.05 μg/kg, 0.125 μg/kg, 0.2 μg/kg, and 0.5 μg/kg in rice, barley, wheat, and oats respectively (Tang et al., 2020).

**Fig. 4:**
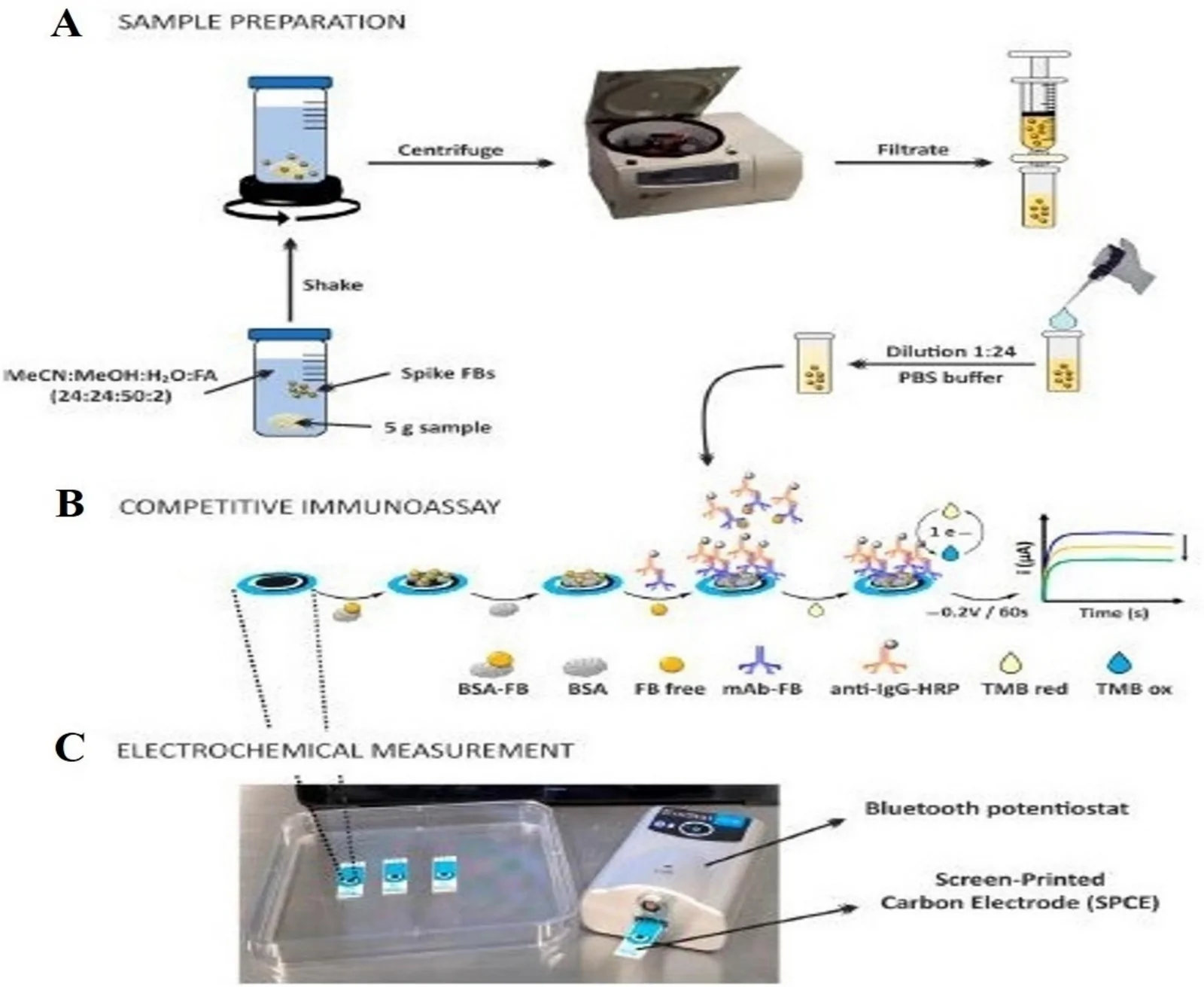
Scheme of (a) maize sample preparation for FB (fumonisins B1 and B2) determination; (b) competitive immunoassay for total FBs on screen-printed carbon electrodes (SPCEs); (c) picture of the setup used for electrochemical measurements via Bluetooth connection (Pérez-Fernández et al., 2025 under Creative Commons Attribution (CC BY) license). MeCN: acetonitrile; MeOH: methanol; PBS: phosphate-buffered saline; BSA: bovine serum albumin; mAb-FB: monoclonal antibody specific to fumonisin; IgG-HRP: polyclonal rabbit anti-mouse horseradish peroxidase labelled antibody; TMB: 3,3′,5,5′-tetramethylbenzidine.

### 7.2 Fruits and vegetables (F & V)

#### 7.2.1 Pesticide residues

A dual-emission carbon dots-based fluorescent immunosensor has been designed for identifying the pesticides carbofuran and atrazine in fruit and vegetable samples by Arsawiset et al. (2025). The immunosensor used green-emitting and red-emitting carbon dots as fluorescent probes. Furthermore, it was reported to be precise, and the LOD of the immunosensor was 0.08 ng/g for atrazine and 0.098 ng/g for carbofuran, which are lower than the MRLs of these pesticides determined by the EU. Furthermore, the immunosensor had recovery rates between 96% and 104% for atrazine, and between 98% and 105% for carbofuran in various spiked fruit and vegetable samples such as apple, basil, cabbage, cauliflower, grape, kale, long bean, morning glory, strawberry, and tomato (Arsawiset et al., 2025). Furthermore, the designed immunosensor retained 89% of its initial fluorescence response intensity even after 8 months of storage, indicating good long-term stability. This long-term stability demonstrates the excellent photostability of CDs. A multicolor immunoassay that could allow naked-eye semiquantitative analysis of isocarbophos in cucumber, orange, and lettuce through a visible color transition from orange to green and blue in response to isocarbophos concentration has been developed by Chen et al. (2024). The multicolour immunoassay reported recovery rates of 89–92.1% in spiked cucumber, 83.3–92.3% in spiked lettuce, and 93.5–95.9% in spiked orange samples (Chen et al., 2024). In another study, an electrochemical immunosensor modified by nanomaterial was developed for detecting parathion in fruit and vegetable samples. The LOD of the immunosensor was determined to be 2.26 pg/mL with a recovery rate within the range of 89.15–116.13% (Yin, Zhang, et al., 2023) (Table 6). The regenerated immunosensor retained more than 90% of its initial activity after the initial two regeneration cycles, approximately 85% after three cycles, and below 75% after four cycles. These results indicate that the immunosensor remained capable of detecting different concentrations of parathion after three regeneration cycles. Furthermore, regarding storage stability, the response of the original immunosensor was considered 100%, and it retained nearly 100% of its initial response after three weeks, approximately 90% after six weeks, and 85% after nine weeks of storage (Yin, Zhang, et al., 2023). Similarly, Talan et al. (2018) developed an electrochemical immunosensor consisting of fluorine-doped tin oxide and gold nanoparticles for detecting chlorpyrifos where the AuNPs helped in improving the sensitivity of the immunosensors by increasing the electrical conductivity (Fig. 5). In that study it was reported that the nano-immunosensor had LOD of 10 fM and could detect chlorpyrifos up to 10 nM in apple and cabbage samples, and till 50 nM for pomegranate samples (Talan et al., 2018). The nano-immunosensor was stored at 4 °C for up to 21 days and exhibited no significant change in peak current during storage. A minimal decrease in peak current was observed after 20 days, confirming the sensor's good storage stability (Talan et al., 2018). In another study, Dai et al. (2017) designed a gold-coated microcantilever-based immunosensor for detecting carbofuran in Chinese chives, tomatoes, and cabbage samples with a low detection limit of 0.1 ng/mL and high specificity. The repeatability of the microcantilever-based immunosensor was evaluated using carbofuran-spiked samples at a concentration of 5.0 × 10^−5^ g/L. Five consecutive measurements using the same immunosensor yielded a relative standard deviation (RSD) of 4.9%. In addition, five independently fabricated immunosensors prepared using the same method showed an RSD of 5.7% for the same carbofuran concentration. These low RSD values suggest the good repeatability of the developed immunosensor (Dai et al., 2017).

**Table 6:** Detection of pesticide residues in fruits and vegetables (F & V) using an immunosensor.

Country of study	Type of sensor	Type of nanomaterials used	Type of antibody used	Targeted pesticide residue	Type of F & V	Linear range	Detection limit	Stability and reproducibility	References
China	Electrochemical	Screen-printed electrode, cobalt (II, III) oxide nanoparticles	Anti-diethoxythiophosphate organophosphorus (polyclonal)	Parathion, quinalphos, coumaphos, and triazophos	Apple; tomato, and spinach (all spiked)	1–10^4^ ng/mL (for each pesticide)	0.416 ng/mL (parathion); 0.298 ng/mL (quinalphos); 0.300 ng/mL (coumaphos); 0.411 ng/mL (triazophos)	Good stability and reproducibility	Wang et al., 2025
India	Electrochemical	Graphene oxide (GO) layered electrode	Anti- monocrotophos (monoclonal)	Monocrotophos	Cabbage	1 ppt − 1 ppm	0.49 ppm	4 weeks and 4 times	Shrikrishna et al., 2024
China	Electrochemical	Glassy carbon electrode, gold nanoparticles (AuNPs), and graphene oxide	Anti-pyrethroid (monoclonal)	Fenpropathrin and deltamethrin	Lettuce, and baby cabbage (all spiked)	0.286–0.286 × 10^5^ nM for fenpropathrin; 1.979–1.979 × 10^5^ nM for deltamethrin	0.0258 nM for fenpropathrin; 1.712 nM for deltamethrin	Up to 336 h and good reproducibility	Xiang et al., 2022
China	Optical	CM7 chip	Anti- triazophos (monoclonal)	Triazophos	Appel; Chinese cabbage, and cucumber (all real samples)	0.98–8.29 ng/mL	0.096 ng/mL	>160 cycles and NR	Guo et al., 2018
Japan	Optical	CM5 chip	Anti- boscalid (monoclonal)	Boscalid	Orange; cucumber, tomato, green sweet pepper, cabbage, and spinach (all spiked)	17–80 ng/mL	0.87 mg/kg	NR	Hirakawa et al., 2015
Spain	Electrochemical	Graphite−epoxy composite electrodes	Anti- paraquat	Paraquat	Potato (spiked)	6.63–105.55 μg/kg	2.18 μg/kg	NR	Garcia-Febrero et al. (2013)
China	Electrochemical	Gold electrode	Anti- Dichlorophenoxybutyric acid (monoclonal)	Dichlorophenoxybutyric acid	Soybean (spiked)	1.0 × 10^−7^ − 1.0 × 10^−3^ g/L	1.0 × 10^−7^ g/L	Up to 4 months and good reproducibility	Zhang et al. (2012)

NR: Not reported.

**Fig. 5:**
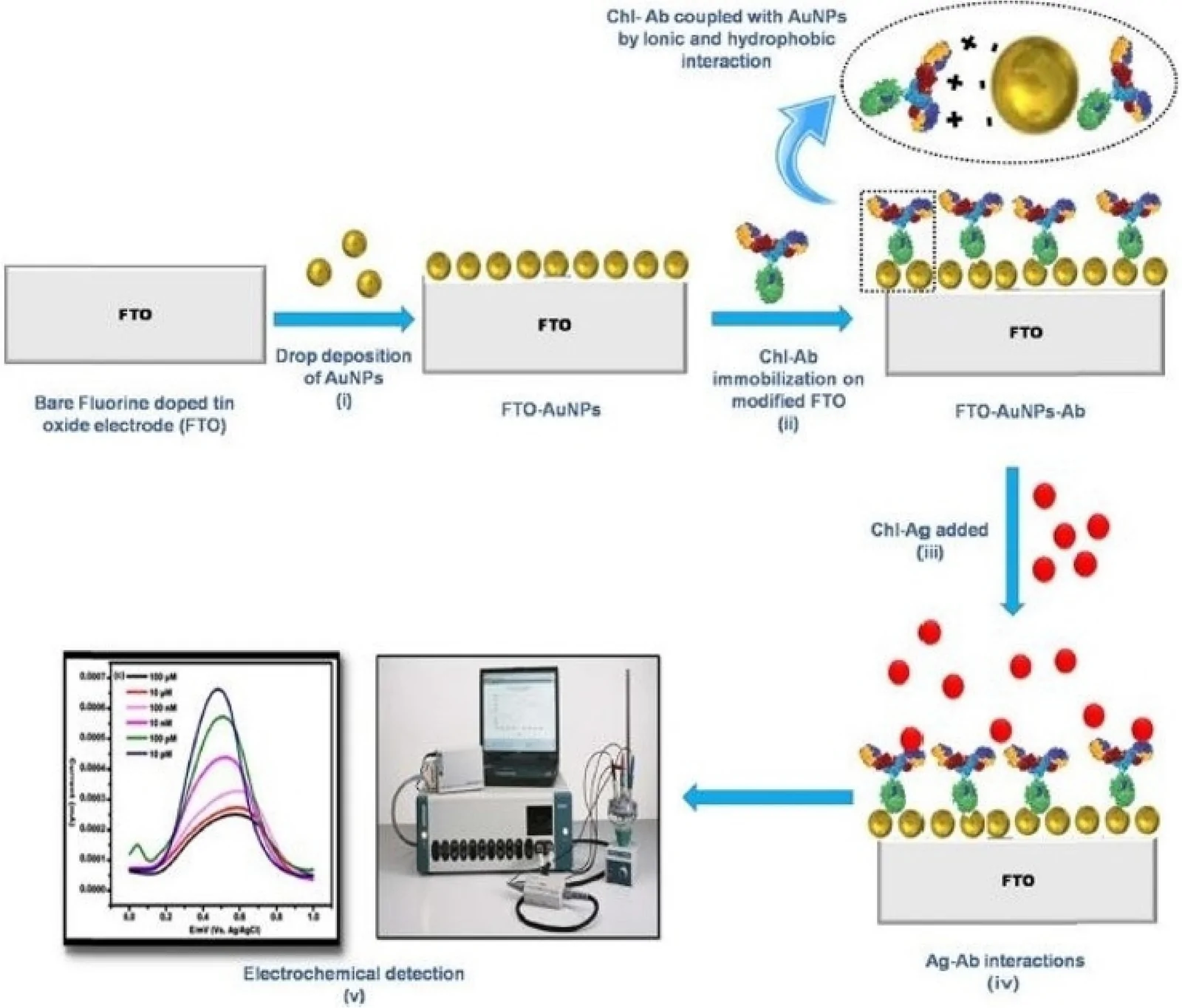
Schematic representation of the fabrication process of FTO-AuNPs-chl-Ab where (i) bare FTO electrode was used and (ii) AuNPs were drop casted on its surface. Anti-chl-Abs were immobilized on FTO-AuNPs modified surface and (iv) Ag-Ab interactions were analyzed by (v) Electrochemical detection (Talan et al., 2018 with license number 6275931156496).

Most of the immunosensors discussed in this review for detecting mycotoxins and pesticides in fruits, vegetables, cereals, and cereal-based products are based on electrochemical approaches. A previous review by Felix and Angnes (2018) highlighted that the key advantages of electrochemical immunosensors are high sensitivity and selectivity, the possibility of multiplexing and portability, and miniaturization, which have contributed to their growing interest and adoption in recent years. Furthermore, the relatively low-cost of developing these immunosensors can provide an additional advantage in various situations. Some of the immunosensors discussed in this review are based on the resonance energy transfer (RET) mechanism, with fluorescence resonance energy transfer (FRET) being the most commonly utilized approach. FRET-based immunosensors were widely reported to use fluorescent dyes and fluorescent proteins (FPs). In addition, quantum dots (QDs), carbon nanotubes, graphene oxide, carbon dots (CDs), and metal nanoparticles (MNPs) have also been used in these sensors (Wu & Jiang, 2022). Over the years, rapid advances in the field of nanotechnology have provided new opportunities to improve the performance of the immunosensors. The high surface-to-volume ratio of nanomaterials facilitates the immobilization of more biorecognition molecules on electrode surfaces, thereby improving the sensitivity and overall performance of these sensors (Adabi et al., 2025). Moreover, these nanomaterials promote rapid electron transfer and enhance the electrode's electrocatalytic activity while reducing the electrode–electrolyte interface impedance (Police Patil et al., 2023).

## 8 Limitations of antibody-based sensing devices and strategies to address them

Relatively low sensitivity and accuracy are among the major limitations of conventional ELISA that have restricted its broader application in food contamination analysis. Consequently, considerable efforts have been devoted to developing strategies to overcome these limitations. In recent years, substantial progress has been made in improving ELISA sensitivity, accuracy, and stability. The development of nanomaterial-based ELISA (nano-ELISA) has improved the analytical performance of conventional ELISA (Wu et al., 2019). Various nanomaterials like molecularly imprinted polymers (MIPs), nanofibers, and magnetic nanobeads have been applied to modify the ELISA adsorbent substrate. Additionally, nanobodies and aptamers have emerged as promising alternatives to conventional recognition elements and can further improve target recognition. Furthermore, nanomaterials that possess peroxidase-like activity, such as gold–platinum bimetallic nanoparticles supported on or encapsulated within a silicon dioxide (silica) shell (Au–Pt@SiO₂), graphene oxide, cerium(IV) oxide nanoparticles, and cupric sulfide nanosheets, have also been widely employed as enzyme labels in nano-ELISA (Dutta et al., 2015; Wu et al., 2017; Zhao, Dong, et al., 2015).

Despite its advantages, LFIA also has several limitations. First, its applicability is restricted to certain types of food contaminants, as detection of some simple-structured contaminants using LFIA remains challenging. For instance, a reliable LFIA-based method for arsenic detection has not yet been established (Wang et al., 2024). Second, developing LFIA requires preparing and optimizing several components, such as specific antibodies, nanoparticles, nitrocellulose membranes, and other assay materials, which can make fabrication relatively complex (Wang et al., 2024). To overcome these limitations, researchers have explored strategies to optimize and enhance LFIA-based detection, such as developing novel labeling materials, optimizing assay parameters, and integrating computational programs. These technological advancements have substantially improved the LFIA accuracy, sensitivity, and stability. Third, although multi-analyte detection platforms have been developed to enable rapid screening of multiple food contaminants, their throughput remains relatively limited, and their quantitative performance is still inferior to that of conventional instrumental analytical methods (Wang et al., 2024). Therefore, improving the accuracy and throughput of LFIA remains an important goal for future development. Further advances could be achieved by integrating LFIAs with digital and smart technologies such as smartphone-based readers, artificial intelligence (AI), and cloud connectivity (Parolo et al., 2020; Sena-Torralba et al., 2022). The integration of these technologies into LFIA can significantly improve accuracy, sensitivity, and accessibility. Furthermore, these advances can facilitate remote and real-time analysis, automate the result analysis, and minimize subjective human errors (Sena-Torralba et al., 2022).

The stability of antibodies plays an important role in determining their affinity and specificity. Hence, it is important to preserve antibodies and maintain proper storage conditions to ensure the reliability and accuracy of antibody-based sensing platforms for detecting pesticides and mycotoxins in fruits, vegetables, cereals, and cereal-based products (Ma et al., 2020). Antibodies can also undergo aggregation during freeze-thaw cycles because of physical and chemical changes occurring in the solute and solvent environment (Desai et al., 2017). Furthermore, different stress factors such as ice crystal formation, crystallization of buffers, gas-liquid interfaces formed due to rapid cooling, cold denaturation, pH changes, and redistribution of solutes that can occur during repeated freeze-thaw cycles can affect the activity of enzyme-conjugated antibodies (Kumar et al., 2025; Laptoš & Omersel, 2018). Hence, to maintain the functionality of the antibodies, it is important to decrease the number of freeze-thaw cycles and use cryoprotectants to prevent aggregation due to the freeze-thaw process (Ma et al., 2020). Furthermore, the use of excipients and optimized buffers can help maintain the long-term stability and potency of the antibodies following multiple freezing and thawing cycles (Chavez et al., 2016; Laptoš & Omersel, 2018).

Furthermore, nanobodies derived from camelids can serve as recognition elements for overcoming stability issues in antibody-based sensing devices. Moreover, the small size and strong binding affinity of nanobodies help them retain functionality across varying pH levels during denaturation and renaturation cycles (Jin et al., 2023; Singh et al., 2016). A study reported that the nanobody-based immunosensor had a prolonged shelf life and remained stable at temperatures as high as 40 °C, where traditional antibodies denature and lose their function (Singh et al., 2016). These properties suggest that nanobodies may be more suitable for antibody-based sensing applications than traditional polyclonal or monoclonal antibodies, especially in on-site applications where antibodies are exposed to harsh environmental conditions and in resource-limited environments where refrigeration may not be available. Moreover, it has been suggested that integrating nanobodies and nanomaterials into antibody-based sensing devices such as immunosensors can help in improving the performance of the sensing devices (Bastos-Soares et al., 2020). Furthermore, combining nanomaterials with nanobodies can help maintain nanobody affinity and specificity and improve the sensitivity and performance of pesticide and mycotoxin sensing devices in fruits, vegetables, and cereals (Bastos-Soares et al., 2020).

Another challenge in the detection of pesticides and mycotoxins in fruits, vegetables, cereals, and cereal-based products using antibody-based sensing devices is the matrix effect. The matrix effect can be defined as alterations in the analytical signal because of the sample matrix and co-eluting impurities present with the target analyte. The matrix effect may cause signal suppression or enhancement, thereby affecting the accuracy of the sensing device. Several compounds that occur naturally in fruits, vegetables, and cereals, such as enzymes, lipids, minerals, pigments, proteins, and sugars, can interfere with the performance of antibody-based sensing methods (Kim, Lee, et al., 2023). A study by Liu, Qin, et al. (2021) and Liu, Xu, et al. (2021) has reported that the use of 30% methanol for extracting metsulfuron-methyl from food samples was more efficient at minimizing matrix effects. They suggested that the matrix effects can be further diminished by diluting the samples. Cereals are complex matrices composed of proteins, lipids, polysaccharides, sugars, vitamins, and various other macro- and micronutrients that can affect analytical results. A study reported that matrix interference in cereals can be diminished by adding hexane during the extraction process to remove nonpolar compounds, followed by dilution with borax buffer, which reduces the effects of co-extracted substances by precipitation (Boroduleva et al., 2017). Additionally, the use of QuEChERS during pretreatment of vegetable and fruit samples has been recommended to reduce matrix effects (Zhao, Hu, et al., 2015).

## 9 AI-based sensing technologies in food sector: A future roadmap

It is important to differentiate between the two major AI paradigms relevant to this field namely, connectionist and symbolic approaches, before discussing specific sensing applications. Most studies have adopted connectionist methods: machine learning and deep neural networks, which are well suited for identifying and learning complex patterns from raw sensor data (Ding et al., 2025). However, a comprehensive assessment should also consider the contribution of symbolic AI. Symbolic approaches such as rule-based expert systems and ontologies use explicit knowledge representation and human-interpretable logic. Unlike connectionist models, which function as “black boxes,” symbolic AI offers transparent and explainable decision-making frameworks, making it particularly valuable for compliance with regulatory guidelines, decision support, and traceability within food safety systems (Kalmykov & Kalmykov, 2025). In traceability workflows, connectionist models can analyze large volumes of sensor and transaction data to detect subtle patterns, trends, and anomalies. Concurrently, symbolic AI can apply explicit rules and interpret the identified information to ensure that the resulting knowledge is actionable, transparent, and comprehensible to relevant stakeholders (Zhan et al., 2026). Organizations can combine pattern-recognition abilities of the connectionist models with the reasoning and interpretability of symbolic AI to attain better predictive accuracy with operational transparency, which can improve the reliability and effectiveness of food safety systems (Zhan et al., 2026). For example, ontologies like FoodOn provide a standardized global vocabulary that can facilitate automated reasoning across interconnected datasets to trace food items from production through consumption, supporting quality control and enabling rapid investigation of food-borne disease outbreaks (Dooley et al., 2018). Additionally, various frameworks have been developed to regulate track-and-trace data across different stakeholders within agricultural and food supply chains, thereby improving the effectiveness and reliability of responses to food safety incidents (Ameri et al., 2022). Food-sensing prototypes have demonstrated promising performance upon integration with AI-based technologies (Zhan et al., 2026). The integration of different machine learning algorithms with sensors for various processed materials can yield varied outcomes.

## 10 Conclusion

Indirect competitive ELISA has become more successful in monitoring food quality and safety. The incorporation of gold nanoparticles in LFIA plays a major role in enhancing visualization. The majority of the immunosensors used for detecting pesticides and mycotoxins in vegetables, fruits, and cereals are based on electrochemical and optical transducers. In immunosensors, the use of different types of nanomaterials has helped in improving their sensitivity and achieving lower LODs. Food matrix effects and instability during long-term storage remain major challenges for the antibody-based sensing platforms. However, buffer selection can help address this problem to a certain extent. The integration of AI with antibody-based sensing approaches offers future possibilities for improving their function and can also help optimize the analyte extraction from complex food matrices.

## Declaration of AI and AI-assisted technologies in the re-writing process

Not applicable.

## CRediT authorship contribution statement

**Harsh Kumar:** Writing – original draft, Visualization, Validation, Formal analysis. **Shivani Guleria:** Writing – review & editing, Visualization, Investigation. **Lukas Peter:** Writing – review & editing, Visualization, Supervision, Project administration. **Nahid Akhtar:** Writing – review & editing, Visualization. **Rabia Asghar:** Writing – review & editing, Visualization, Validation. **Hasnita Binti Che Harun:** Writing – review & editing, Visualization, Supervision, Project administration. **Dinesh Kumar:** Writing – review & editing, Visualization. **Kamil Kuča:** Writing – review & editing, Visualization, Supervision, Project administration. **Ayaz Mukarram Shaikh:** Writing – review & editing, Visualization, Validation, Software, Resources. **Endre Harsányi:** Writing – review & editing, Visualization, Validation, Supervision, Project administration, Methodology, Funding acquisition. **Béla Kovács:** Writing – review & editing, Visualization, Resources, Project administration, Funding acquisition.

## Funding

The funding is supported by the University of Debrecen Program for Scientific Publication.

## Declaration of competing interest

The authors declare that they have no known competing financial interests or personal relationships that could have appeared to influence the work reported in this paper.

## Data Availability

No data was used for the research described in the article.
